# The Potential Link and Role of Zyxin in the Pathogenesis of Psoriasis and Its Associated Comorbidities

**DOI:** 10.3390/ijms27020639

**Published:** 2026-01-08

**Authors:** Mateusz Matwiejuk, Agnieszka Kulczyńska-Przybik, Bartłomiej Łukaszuk, Hanna Myśliwiec, Piotr Myśliwiec, Adrian Chabowski, Barbara Mroczko, Iwona Flisiak

**Affiliations:** 1 Department of Dermatology and Venereology, Medical University of Bialystok, 15-089 Białystok, Poland; 2 Department of Neurodegeneration Diagnostics, Medical University of Bialystok, 15-269 Bialystok, Poland; 3 Department of Physiology, Medical University of Bialystok, 15-222 Bialystok, Poland; 4 1st Clinical Department of General and Endocrine Surgery, Medical University of Bialystok, 15-276 Bialystok, Poland

**Keywords:** psoriasis, skin diseases, protein, zyxin

## Abstract

Psoriasis is a chronic inflammatory disorder with immunological, metabolic, and environmental components. It affects not only the skin but also the nails, joints, and vascular system. A total of 50 patients with psoriasis and 28 healthy controls took part in this study. Serum samples were gathered both from the psoriatic group and the control group. Serum zyxin concentrations were measured via enzyme-linked immunosorbent assay (ELISA). Our results revealed that serum zyxin amounts were significantly higher in patients with psoriasis compared with the controls. However, no statistically significant correlations were found between serum zyxin levels and inflammatory or metabolic parameters in the psoriasis group. Similarly, there was no significant correlation between zyxin level and disease severity as assessed by the Psoriasis Area and Severity Index (PASI) score. To sum up, our study demonstrates that serum zyxin levels are significantly elevated in patients with psoriasis compared with controls. Nevertheless, the precise role of zyxin in the aetiology of psoriasis remains unclear. Further research is needed to clarify the function of this protein in the disease process and to explore its potential as a therapeutic target.

## 1. Introduction

Psoriasis is a chronic, complex, and inflammatory dermatosis caused by a combination of genetic, environmental, and immunological factors [1]. According to the World Psoriasis Day consortium, 125 million people worldwide, comprising 2–3% of the global population, currently suffer from psoriasis [2]. In contrast to the previously mentioned data, the WHO [3] has revealed that circa 0.84% (0.09% to 11.43%) of the global population (about 64.6 million people) are currently dealing with psoriasis [3]. Psoriasis affects men and women with nearly equal prevalence [4]. The coexistence of psoriasis with multiple comorbidities can lead not only to a physical burden, but also to an economic one. Psoriasis is frequently linked to the development of cardiovascular diseases (CVD) (such as coronary artery disease, dysrhythmias, heart failure, and vascular disease) [5,6], metabolic syndrome [7,8], inflammatory bowel disease (Crohn’s disease and ulcerative colitis) [9,10,11], uveitis [12,13], and mental disorders (such as depression, bipolar disorder, anxiety, schizophrenia, and suicidal ideation) [14,15,16,17,18]. The pathogenesis of psoriasis is very complex; however, due to progress in clinical research, it is becoming increasingly more understood [19]. Various types of cells are key in the pathological mechanisms of psoriasis: keratinocytes, T cells, myeloid dendritic cells, plasmacytoid dendritic cells, macrophages, and neutrophils all play a part in the inflammatory process and progression of the disease [20].

One interesting, yet not yet fully assessed, protein involved in the pathogenesis of psoriasis is zyxin [21]. Zyxin is a cytoskeletal protein whose fragments are present within the blood [22]. More specifically, zyxin is part of the LIM protein group. It is characterized by a proline-rich ActA-repeat region, leucine-rich nuclear export sequence (NES), serine phosphorylation features, and cysteine/histidine-coordinated LIM domains [23]. Interestingly, the N-terminal part of zyxin possesses special binding sites for actin and vasodilator-stimulated phosphoprotein, which handle the regulatory function of actin [24,25]. Looking at the cellular level, zyxin is a key participant in stretch-induced gene expression. The absence of this protein may cause delays in the strengthening of vascular smooth muscle cells due to amplified stretch [26]. Beyond zyxin’s impact on endothelial cell exocytosis, it may also regulate mechanosensitive gene expression and enhance the adhesion and migration of tumour cells [27,28,29]. As psoriasis is treated as a generalized inflammation disorder with a higher cardiovascular risk, evaluating serum zyxin levels may offer insight into shared pathogenic mechanisms and their potential role as biomarkers of systemic involvement.

So far, there are limited data on the function of zyxin in the pathogenesis of psoriasis. In the first study we conducted, zyxin levels were found to be higher in plasma from the psoriatic group in comparison to the healthy group [21]. In the study conducted by Kim et al. [30], zyxin levels were elevated in the serum of patients with psoriasis and CVD compared to the serum of patients from the control group [30]. However, it has been shown that zyxin plays a role in various cellular processes, such as migration, mechanotransduction, cell adhesion, and the regulation of signalling cascades through the TGF-β and YAP/TAZ pathways. These processes are related to tissue remodelling and inflammatory states, which are characteristic features of psoriasis [31].

The goal of this research was to elucidate the function of zyxin in the pathogenesis, development, and progression of psoriasis. In this study, we evaluated serum zyxin levels in patients with psoriasis and compared them with those of healthy controls. Moreover, we examined the associations between serum zyxin levels and disease severity assessed by the Psoriasis Area and Severity Index (PASI) score, as well as with selected biochemical, inflammatory, and clinical parameters.

## 2. Results

In this study, 50 patients with active plaque psoriasis (20 females, 30 males) and 28 controls (24 females, 4 males) were enrolled. The median age was 42 years (interquartile range [IQR]: 37.5–47.2 years) in the control group and 51 years (IQR: 34.2–66.0 years) in the psoriasis group. The median length of psoriasis was 16 years. In the healthy group, median body weight was 69.5 kg (IQR: 63.8–79.2 kg), mean height was 165.0 cm (IQR: 161.5–170.2 cm), and median body mass index (BMI) was 25.1 kg/m^2^ (IQR: 23.5–27.9 kg/m^2^). According to BMI categories, 12 participants (43%) had normal weight (18.5–24.9), 11 (39%) were overweight (25.0–29.9), and 5 (18%) were obese (>30.0). In the psoriasis group, median body weight was 84.0 kg (IQR: 75.5–95.8 kg), mean height was 172.5 cm (IQR: 164.2–176.0 cm), and median BMI was 29.0 kg/m^2^ (IQR: 23.9–31.8 kg/m^2^). Among these patients, 14 (28%) had normal weight, 17 (34%) were overweight, and 19 (38%) were obese. Regarding the disease severity, 7 patients (14%) had mild psoriasis (PASI < 10), whereas 43 patients (86%) had moderate-to-severe psoriasis (PASI ≥ 10). Of the latter, 26 patients (52%) had a PASI score of 10–20, and 17 patients (34%) had a PASI score > 20.

### Zyxin Parameter

**Figure 1:**
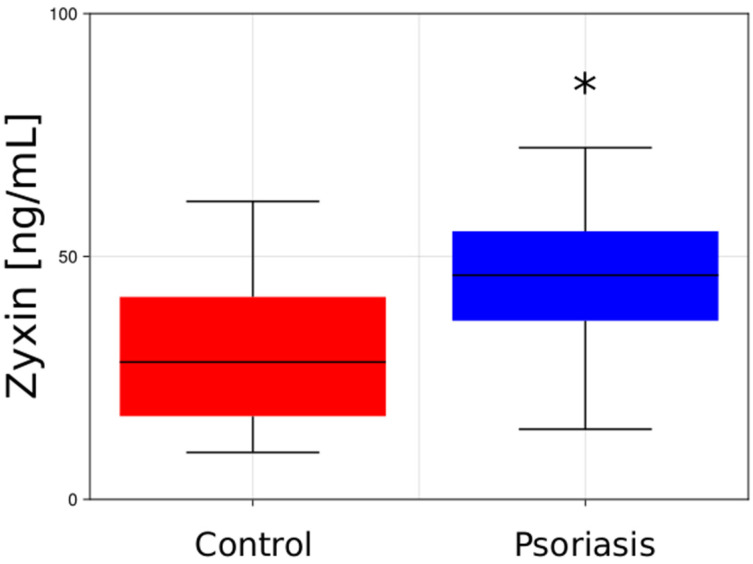
Comparison between the zyxin level in the serum from the control patients and the serum from the psoriatic patients [ng/mL]. *—different vs. control group (*p* < 0.05).

The median level of zyxin observed in the serum of psoriatic patients (46.1 [ng/mL]) was revealed to be significantly higher (*p* < 0.05) compared to the control group (28.2 [ng/mL]). Figure 1 illustrates the varying zyxin levels in both the healthy individuals and in the patients with psoriasis.

## 3. Discussion

In our article, we evaluated serum zyxin levels in psoriatic patients compared to controls. Additionally, we assessed the relationship between serum zyxin levels and both laboratory parameters and clinical features in patients with psoriasis.

We noticed that the amount of zyxin was significantly higher (*p* < 0.05) in the serum of psoriatic patients in comparison to the controls.

**Figure 2:**
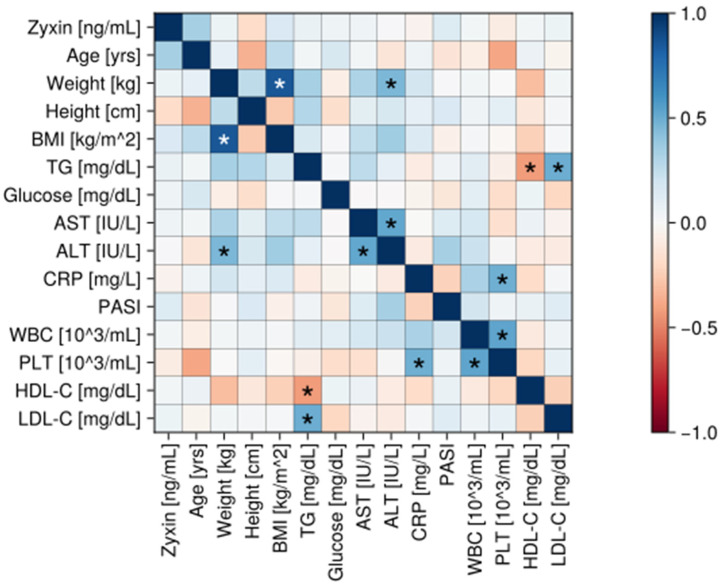
Correlation matrix (heatmap) in the psoriatic group. Pearson correlation coefficients are depicted as shades of blue (positive correlation) or red (negative correlation). BMI—body mass index, CRP—C-reactive protein, HDL-C—high-density lipoprotein, LDL-C—low-density lipoprotein, TG—triacylglycerol, AST—Aspartate transaminase, ALT—Alanine transaminase, WBC—white blood count, PLT—platelet count, PASI—Psoriasis Area Severity Index. *—statistically significant correlation (*p* < 0.05).

In the Figure 2, although we observed both positive and negative correlations between zyxin and age (r = 0.39; *p* = 0.058), zyxin and height (r = −0.22; *p* = 0.4528), zyxin and weight (r = 0.02; *p* = 0.9771), zyxin and BMI (r = 0.14; *p* = 0.8325), zyxin and TG (r = 0.12; 0.88250), zyxin and LDL-C (r = 0.1; *p* = 0.8825), zyxin and HDL-C (r = 0.05; *p* = 0.9771), zyxin and WBC (r = 0.11; 0.8825), zyxin and PLT (r = −0.1; 0.8825), zyxin and AST (r = 0.09; *p* = 0.8825), zyxin and ALT (r = 0.04; *p* = 0.9771), zyxin and glucose (r = 0.06; *p* = 0.9647), and zyxin and CRP (r = −0.02; *p* = 0.9771), none reached the level of statistical significance.

Similarly to our results, in their research, Plavina et al. [21] observed a higher amount of zyxin in the plasma of psoriatic patients in comparison to healthy patients [21].

Kim et al. [30] reported higher serum zyxin levels in patients with psoriasis compared to healthy controls. The authors suggested that zyxin may serve as a biomarker associated with CVD risk factors, including alcohol consumption, diabetes mellitus, dyslipidemia, hypertension, obesity, and smoking [30].

However, contrary to these outcomes, we did not observe any association between serum zyxin amount and CVD risk factors in our cohort. This discrepancy may be explained by the absence of psoriatic patients with concomitant cardiovascular disease in our study population.

So far, there are limited published articles on the role of zyxin in the pathogenesis of psoriasis. In our study, we demonstrated only a statistically significant increase in zyxin amounts in the serum of patients with psoriasis in comparison to the controls. Zyxin may well have an extended role in the general psoriatic pathogenesis, but this is a role that has not yet been precisely clarified.

Furthermore, we investigated potential correlations between zyxin levels and various metabolic parameters in the serum of patients suffering from psoriasis, as well as correlations with psoriasis severity, in comparison to healthy individuals. However, these results did not reach statistical significance.

Although the observed trends suggest a possible association between zyxin levels and psoriasis, as well as its related comorbidities, none of the correlations analyzed statistical significance and should therefore be interpreted with caution.

A tendency toward higher zyxin levels with increasing age was observed in patients with psoriasis (zyxin and age, r = 0.39; *p* = 0.058). While psoriasis may occur at any age and typically presents in two incidence peaks (20–30 and 50–60 years), older patients with psoriasis more frequently develop comorbid conditions, particularly cardiovascular disease and diabetes. In this context, elevated zyxin levels may reflect an increased burden of age-related comorbidities rather than an age-dependent risk of psoriasis onset itself. No meaningful association was observed between zyxin levels and glucose concentration (zyxin and glucose, r = 0.06; *p* = 0.9647) [32].

A weak positive correlation was also noted between zyxin levels and BMI in the psoriasis group (r = 0.14; *p* = 0.8325). Although not statistically significant, this trend may be of interest given that overweight and obesity are common comorbidities in patients with psoriasis and share underlying inflammatory mechanisms [33,34]. Chronic systemic inflammation in psoriasis promotes keratinocyte hyperproliferation through the activation of Th1, Th17, and Th22 lymphocytes and increased production of pro-inflammatory cytokines, such as IL-1, IL-6, IL-12, IL-23 and TNF-α [35,36].

Furthermore, zyxin levels showed weak positive correlations with lipid parameters, including triglycerides (zyxin and TG, r = 0.12; *p* = 0.8825) and LDL-cholesterol (zyxin and LDL-C, r = 0.10; *p* = 0.8825). These findings may suggest a potential link between zyxin and metabolic disturbances, such as dyslipidemia, which is known to occur more frequently in patients with psoriasis. Such patients have an approximately 1.4-fold-increased risk of developing metabolic disorders compared with healthy individuals [37,38].

Finally, weak positive correlations were observed between zyxin levels and liver enzymes (zyxin and AST, r = 0.09; *p* = 0.8825; zyxin and ALT, r = 0.04; *p* = 0.9771). Although these associations were not significant, they may reflect the coexistence of psoriasis with liver disorders, including non-alcoholic fatty liver disease, which has been increasingly recognized as a psoriasis-related comorbidity [39,40,41,42].

Altered zyxin expression has also been reported in several human malignancies, supporting its involvement in pathological processes beyond inflammatory skin disease. In cases of breast cancer, zyxin protein levels have been found to be significantly higher in tumour tissues compared with adjacent normal breast tissue, with moderate to high expression observed in over 70% of tumour samples. Moreover, increased zyxin expression has been associated with advanced histological-stage and lymph node metastasis, suggesting a potential role in tumour progression and as a prognostic marker [43]. Similarly, elevated zyxin levels have been reported in both colorectal cancer and hepatocellular carcinoma, where they contribute to cytoskeletal remodelling, cell migration, and proliferation [44,45]. These findings indicate that zyxin may be involved in diseases which are characterized by altered cell adhesion, migration, and tissue remodelling.

Psoriasis is associated with a modestly increased overall risk of malignancy, particularly for keratinocyte cancers, lymphomas, lung cancer, and bladder cancer, although the evidence for increased incidence of specific solid tumours remains inconsistent [46]. Therefore, although zyxin levels are elevated in certain cancers, a direct link between increased zyxin levels in psoriasis and cancer risk cannot currently be established. Elevated zyxin levels in psoriasis may instead reflect shared underlying mechanisms, including chronic inflammation, enhanced cellular migration, and altered mechanotransduction. Subsequently, zyxin may contribute to accelerated epidermal cell turnover, hyperkeratosis, acanthosis, and dilatation of blood vessels in the psoriatic skin. Moreover, in the future, this protein could be a key target for various drugs, for instance, as a topical, systemic treatment, including biological treatment, to inhibit inflammation pathways.

Based on our findings and the current level of knowledge, the precise function of zyxin in the aetiology of psoriasis, and its potential association with components of metabolic syndrome, remains unclear. Further investigation is needed to more accurately elucidate the possible route of the psoriatic aetiology and the involvement of zyxin therein.

### Limitations

The present study has several limitations. The study group and healthy group were not fully linked for age, sex, and BMI, resulting in a higher cardiovascular risk profile in patients with plaque psoriasis. As cardiovascular disease has been associated with increased zyxin concentrations, this imbalance may have acted as a confounding factor, and the observed differences cannot be attributed solely to psoriasis. At the same time, the elevated zyxin levels observed in the psoriasis group may reflect the increased burden of cardiovascular risk factors commonly present in these patients.

In addition, the limited number of participants and the cross-sectional nature limit causal inference. Therefore, the results should be considered as preliminary. Future investigations with larger, well-matched cohorts are required in order to validate these results and to clarify whether zyxin may serve as a biomarker of cardiovascular risk in patients with psoriasis.

## 4. Materials and Methods

In this study, we examined 50 patients (20 females and 30 males) that were affected by active plaque-type psoriasis (median age: 51.0). The individuals were hospitalized in the Department of Dermatology and Venereology, Medical University of Bialystok. The study group included adult patients with plaque psoriasis who had not received any systemic anti-psoriatic treatment, including methotrexate, acitretin, cyclosporine, or biologics) for at least 4 weeks prior to department enrollment. Patients receiving systemic treatment were excluded. The control group comprised 28 patients with inguinal hernia (24 females, 4 males, median age: 42.0) who were admitted to the hospital in the I Clinic of General and Endocrine Surgery, Medical University of Bialystok. This study was based on the same patient data that we reported on in our previous study [47]. Psoriasis severity was calculated using the PASI [48]. Body mass index (BMI) was evaluated using weight and height as reported by the participants [49]. None of the participants, either patients or controls, were subject to dietary restrictions. Histories of chronic conditions, including hypertension, liver disease (such as non-alcoholic fatty liver disease), cardiovascular disease, and diabetes mellitus, as well as relevant laboratory test results, were obtained from hospital records, and individuals with these conditions were excluded from the study. During the study period, neither the study group nor the control group participants were exposed to dietary restrictions, consumed protein-rich supplements or high-protein foods, nor used medications known to affect protein metabolism. All psoriatic patients and healthy volunteers provided written informed consent prior to inclusion in the study. The research protocol was approved by the local university bioethics committee (No. APK.002.272.2025) and was conducted in accordance with the ethical principles of the Declaration of Helsinki. Peripheral blood samples were collected following an overnight fast and before the initiation of any treatment. After centrifugation, serum samples were stored at −80 °C until further analysis.

### 4.1. Zyxin Analysis

The commercially available sandwich-type ELISA kit was applied for the quantification of zyxin protein in the studied samples (ELK Biotechnology, Wuhan, China). ELISAs were conducted following the instructions from the manufacturer. Tested serum samples were diluted in a ratio of 1:5. In the ELISAs, the final volume per well was 100 µL. The sensitivity of the kit allowed for the detection of the tested protein in a range of 0.16–10 ng/mL. The calibration curve was constructed using a four-parameter curve-fitting model. Subsequently, the final serum concentrations of zyxin were quantified and recorded in nanograms per millilitre (ng/mL).

### 4.2. Statistical Analysis

Statistical analyses were performed using Julia version 1.10.8. Between-group comparisons of continuous variables were assessed using Student’s *t*-test when the groups were characterized by normal distributions and homogenous variances. If the above did not hold, the Mann–Whitney U test (also known as the Wilcoxon test) was performed. Categorical variables (such as gender; see Table 1) were analyzed using Fisher’s exact test for categorical data. Correlation analyses (heatmaps) were generated from Pearson’s correlation coefficient, and their *p*-values were adjusted for multiple comparisons (Benjamini–Hochberg correction). The obtained *p*-values < 0.05 were treated to reach the level of statistical significance.

## 5. Conclusions

Our results demonstrate significantly higher serum zyxin levels in patients with psoriasis compared with controls. These findings suggest that zyxin may be involved in systemic processes associated with psoriasis. However, the lack of a significant correlation with psoriasis severity (PASI score), inflammatory markers, or metabolic parameters indicates that the precise role of zyxin in psoriasis remains unclear. Therefore, zyxin should currently be regarded as a potential marker of psoriasis-associated systemic alterations rather than a direct indicator of disease activity. Further studies in larger, well-characterized, and better-matched cohorts are required in order to clarify its biological significance and clinical relevance.

## Figures and Tables

**Table 1:** The main clinical characteristics of the psoriatic group and the control group.

Clinical and Laboratory Features	Control	Psoriasis
**Age [years]**	42.0 (37.5–47.2)	51.0 (34.2–66.0)
**Weight [kg]**	69.50 (63.8–79.2)	84.0 (75.5–95.8) *
**Height [cm]**	165.0 (161.5–170.2)	172.5 (164.2–176.0) *
**BMI [kg/m^2^]**	25.1 (23.5–27.9)	29.0 (23.9–31.8) *
**CRP [mg/dL]**	1.0 (1.0–1.9)	3.2 (1.5–6.9) *
**Glucose [mg/dL]**	86.5 (78.8–91.0)	85.0 (80.0–93.0)
**TG [mg/dL]**	91.5 (73.8–117.8)	116.0 (86.2–134.5)
**AST [U/L]**	17.5 (15.0–21.0)	20.0 (16.2–27.0) *
**ALT [U/L]**	15.5 (11.5–18.2)	19.0 (14.2–27.8) *
**Sex [no. female/no. male]**	24/4	20/30 *

Clinical and biochemical characteristics of the control group and psoriatic patients. Data are presented as median and interquartile range. *—different vs. control (*p* < 0.05); BMI—body mass index, CRP—C reactive protein, TG—triacylglycerol, AST—Aspartate transaminase, ALT—Alanine transaminase.

## Data Availability

The original contributions presented in this study are included in the article. Further inquiries can be directed to the corresponding author.

## Abbreviations

CRP	C-reactive protein
TG	Triacylglycerol
AST	Aspartate transaminase
ALT	Alanine transaminase
WBC	White blood count
PLT	Platelet count
BMI	Body Mass Index
CVD	Cardiovascular diseases
NES	Nuclear export sequence
PASI	Psoriasis Area and Severity Index
NAFLD	Non-alcoholic fatty liver disease

## References

[1] Yamanaka K., Yamamoto O., Honda T. (2021). Pathophysiology of psoriasis: A review. J. Dermatol..

[2] National Psoriasis Foundation. https://www.psoriasis.org/psoriasis-statistics/.

[3] World Health Organization (2016). Global Report on Psoriasis. https://www.who.int/publications/i/item/9789241565189.

[4] Parisi R., Iskandar I.Y.K., Kontopantelis E., Augustin M., Griffiths C.E.M., Ashcroft D.M., Global Psoriasis Atlas (2020). National, regional, and worldwide epidemiology of psoriasis: Systematic analysis and modelling study. BMJ.

[5] Zhang L., Wang Y., Qiu L., Wu J. (2022). Psoriasis and cardiovascular disease risk in European and East Asian populations: Evidence from meta-analysis and Mendelian randomization analysis. BMC Med..

[6] Gao N., Kong M., Li X., Zhu X., Wei D., Ni M., Wang Y., Hong Z., Dong A. (2022). The Association Between Psoriasis and Risk of Cardiovascular Disease: A Mendelian Randomization Analysis. Front. Immunol..

[7] Taliercio M., Lebwohl M. (2024). Psoriasis Comorbidities and Their Treatment Impact. Dermatol. Clin..

[8] Armstrong A.W., Harskamp C.T., Armstrong E.J. (2013). Psoriasis and metabolic syndrome: A systematic review and meta-analysis of observational studies. J. Am. Acad. Dermatol..

[9] Fu Y., Lee C.H., Chi C.C. (2018). Association of Psoriasis with Inflammatory Bowel Disease: A Systematic Review and Meta-analysis. JAMA Dermatol..

[10] Li W.Q., Han J.L., Chan A.T., Qureshi A.A. (2013). Psoriasis, psoriatic arthritis and increased risk of incident Crohn’s disease in US women. Ann. Rheum. Dis..

[11] Augustin M., Reich K., Glaeske G., Schaefer I., Radtke M. (2010). Co-morbidity and age-related prevalence of psoriasis: Analysis of health insurance data in Germany. Acta Derm. Venereol..

[12] Kim B.R., Choi S.W., Choi C.W., Lee K.H., Kim M.J., Woo S.J., Youn S.W. (2023). Risk of uveitis in patients with psoriasis in Korea: A nationwide population-based cohort study. J. Eur. Acad. Dermatol. Venereol..

[13] Chaiyabutr C., Ungprasert P., Silpa-Archa N., Wongpraparut C., Chularojanamontri L. (2020). Psoriasis and Risk of Uveitis: A Systematic Review and Meta-Analysis. Biomed Res. Int..

[14] Hu S.C., Chen G.S., Tu H.P. (2019). Epidemiology of Depression in Patients with Psoriasis: A Nationwide Population-based Cross-sectional Study. Acta Derm. Venereol..

[15] Wang Y., Wang X., Gu X., Pan J., Ouyang Z., Lin W., Zhu W., Wang M., Su J. (2023). Evidence for a causal association between psoriasis and psychiatric disorders using a bidirectional Mendelian randomization analysis in up to 902,341 individuals. J. Affect. Disord..

[16] Hedemann T.L., Liu X., Kang C.N., Husain M.I. (2022). Associations between psoriasis and mental illness: An update for clinicians. Gen. Hosp. Psychiatry.

[17] Dowlatshahi E.A., Wakkee M., Arends L.R., Nijsten T. (2014). The prevalence and odds of depressive symptoms and clinical depression in psoriasis patients: A systematic review and meta-analysis. J. Investig. Dermatol..

[18] Dalgard F.J., Gieler U., Tomas-Aragones L., Lien L., Poot F., Jemec G.B.E., Misery L., Szabo C., Linder D., Sampogna F. (2015). The psychological burden of skin diseases: A cross-sectional multicenter study among dermatological out-patients in 13 European countries. J. Investig. Dermatol..

[19] Griffiths C.E.M., Armstrong A.W., Gudjonsson J.E., Barker J.N.W.N. (2021). Psoriasis. Lancet.

[20] Ogawa E., Sato Y., Minagawa A., Okuyama R. (2018). Pathogenesis of psoriasis and development of treatment. J. Dermatol..

[21] Plavina T., Hincapie M., Wakshull E., Subramanyam M., Hancock W.S. (2008). Increased plasma concentrations of cytoskeletal and Ca2+-binding proteins and their peptides in psoriasis patients. Clin. Chem..

[22] Moon H.S., Even-Ram S., Kleinman H.K., Cha H.J. (2006). Zyxin is upregulated in the nucleus by thymosin beta4 in SiHa cells. Exp. Cell Res..

[23] Cattaruzza M., Lattrich C., Hecker M. (2004). Focal adhesion protein zyxin is a mechanosensitive modulator of gene expression in vascular smooth muscle cells. Hypertension.

[24] Bai H., Zhang Y., Zhang X., Li C., Ma M., Gao J., Deng T., Gao C., Wang N. (2025). Zyxin-a novel detrimental target, is inhibited by Saikosaponin A during allergic asthma. Phytomedicine.

[25] Drees B.E., Andrews K.M., Beckerle M.C. (1999). Molecular dissection of zyxin function reveals its involvement in cell motility. J. Cell Biol..

[26] Ghosh S., Kollar B., Nahar T., Suresh Babu S., Wojtowicz A., Sticht C., Gretz N., Wagner A.H., Korff T., Hecker M. (2015). Loss of the mechanotransducer zyxin promotes a synthetic phenotype of vascular smooth muscle cells. J. Am. Heart Assoc..

[27] Han X., Li P., Yang Z., Huang X., Wei G., Sun Y., Kang X., Hu X., Deng Q., Chen L. (2017). Zyxin regulates endothelial von Willebrand factor secretion by reorganizing actin filaments around exocytic granules. Nat. Commun..

[28] Uemura A., Nguyen T.N., Steele A.N., Yamada S. (2011). The LIM domain of zyxin is sufficient for force-induced accumulation of zyxin during cell migration. Biophys. J..

[29] Wang Y.X., Wang D.Y., Guo Y.C., Guo J. (2019). Zyxin: A mechanotransductor to regulate gene expression. Eur. Rev. Med. Pharmacol. Sci..

[30] Kim N.Y., Back J.H., Shin J.H., Ji M.J., Lee S.J., Park Y.E., Park H.M., Gu M.B., Lee J.E., Kim J.E. (2023). Quantitative proteomic analysis of human serum using tandem mass tags to predict cardiovascular risks in patients with psoriasis. Sci. Rep..

[31] Wu Z., Wu D., Zhong Q., Zou X., Liu Z., Long H., Wei J., Li X., Dai F. (2024). The role of zyxin in signal transduction and its relationship with diseases. Front. Mol. Biosci..

[32] Glazer Levavi S., Maman R., Sherman S., Mimouni D., Pavlovsky L. (2025). Systemic Biologic Treatment for Psoriasis in Elderly Patients. J. Clin. Med..

[33] Jensen P., Skov L. (2016). Psoriasis and Obesity. Dermatology.

[34] Bellinato F., Maurelli M., Geat D., Girolomoni G., Gisondi P. (2024). Managing the Patient with Psoriasis and Metabolic Comorbidities. Am. J. Clin. Dermatol..

[35] Czarnecka A., Zabłotna M., Purzycka-Bohdan D., Nowicki R.J., Szczerkowska-Dobosz A. (2023). An Observational Study of 147 Psoriasis Patients: Overweightness and Obesity as a Significant Clinical Factors Correlated with Psoriasis. Medicina.

[36] Barros G., Duran P., Vera I., Bermúdez V. (2022). Exploring the Links between Obesity and Psoriasis: A Comprehensive Review. Int. J. Mol. Sci..

[37] Uczniak S., Gerlicz Z.A., Kozłowska M., Kaszuba A. (2016). Presence of selected metabolic syndrome components in patients with psoriasis vulgaris. Postep. Dermatol. Alergol..

[38] Alshahrani J.A., Alshahrani A.M., Ali Alshahrani S., Abdullah Alshahrani F., Saeed Matar Alzahrani M., Jaza Albalawi R., Aljunaid M.A. (2023). A Holistic View of Psoriasis: Examining Its Association with Dyslipidemia and Obesity in a Decade-Long Systematic Review and Meta-Analysis. Cureus.

[39] Klujszo E.H., Parcheta P., Witkowska A.B., Krecisz B. (2020). Non-alcoholic fatty liver disease in patients with psoriasis: Therapeutic implications. Postep. Dermatol. Alergol..

[40] Lin Z., Shi Y.Y., Yu L.Y., Ma C.X., Pan S.Y., Dou Y., Zhou Q.J., Cao Y. (2024). Metabolic dysfunction associated steatotic liver disease in patients with plaque psoriasis: A case-control study and serological comparison. Front. Med..

[41] Untaaveesup S., Kantagowit P., Ungprasert P., Kitlertbanchong N., Vajiraviroj T., Sutithavinkul T., Techataweewan G., Eiumtrakul W., Threethrong R., Chaemsupaphan T. (2025). The Risk of Metabolic Dysfunction-Associated Steatotic Liver Disease in Moderate-to-Severe Psoriasis: A Systematic Review and Meta-Analysis. J. Clin. Med..

[42] González Fernández J., Prieto-Torres L., Ara Martín M., Martínez-Domínguez S.J. (2025). MASLD and liver fibrosis in patients with psoriasis receiving IL-17 or IL-23 inhibitors: A systematic review. Ther. Adv. Gastroenterol..

[43] Kotb A., Hyndman M.E., Patel T.R. (2018). The role of zyxin in regulation of malignancies. Heliyon.

[44] Zhong C., Yu J., Li D., Jiang K., Tang Y., Yang M., Shen H., Fang X., Ding K., Zheng S. (2019). Zyxin as a potential cancer prognostic marker promotes the proliferation and metastasis of colorectal cancer cells. J. Cell Physiol..

[45] Cai T., Bai J., Tan P., Huang Z., Liu C., Wu Z., Cheng Y., Li T., Chen Y., Ruan J. (2023). Zyxin promotes hepatocellular carcinoma progression via the activation of AKT/mTOR signaling pathway. Oncol. Res..

[46] Vaengebjerg S., Skov L., Egeberg A., Loft N.D. (2020). Cancer risk in patients with psoriasis: A systematic review and meta-analysis of epidemiological studies. J. Eur. Acad. Dermatol. Venereol..

[47] Matwiejuk M., Kulczyńska-Przybik A., Łukaszuk B., Myśliwiec H., Myśliwiec P., Chabowski A., Mroczko B., Flisiak I. (2025). The Role of Visfatin/NAMPT in the Pathogenesis of Psoriasis. Metabolites.

[48] Wu D., Lu X., Nakamura M., Sekhon S., Jeon C., Bhutani T., Liao W. (2022). A Pilot Study to Assess the Reliability of Digital Image-Based PASI Scores Across Patient Skin Tones and Provider Training Levels. Dermatol. Ther..

[49] Ardiansyah D., Avianto D. (2024). The Implementation of a Body Mass Index (BMI) Calculator in an Android-Based Ideal Body Check and Nutrition Consultation Application. Int. J. Eng. Technol. Nat. Sci..

